# Transcriptome study of oleanolic acid in the inhibition of breast tumor growth based on high-throughput sequencing

**DOI:** 10.18632/aging.203582

**Published:** 2021-10-04

**Authors:** Zhuoran Liang, Ruolan Pan, Xia Meng, Jinxing Su, Yong Guo, Gang Wei, Zhi Zhang, Kan He

**Affiliations:** 1 School of Forestry, Northeast Forestry University, Harbin, Heilongjiang 150040, PR China; 2 Harbin Vocational and Technical College, Harbin, Heilongjiang 150081, PR China; 3 Center for Stem Cell and Translational Medicine, School of Life Sciences, Anhui University, Hefei, Anhui 230601, PR China

**Keywords:** oleanolic acid, breast cancer, RNA-seq, pathway

## Abstract

The function of oleanolic acid (OA) in various types of cancer has been reported frequently, especially for breast cancer. However, the regulation of breast tumor growth in response to OA treatment has not been studied in depth. Here, we first explored the effect of OA treatment on breast tumors *in vitro* and *in vivo* and then used RNA-seq technology to study the effect and molecular mechanism of OA treatment of MCF-7 cells, particularly at the level of functional genomics. The results showed that 40 μM OA treatment could significantly inhibit the proliferation and induce the apoptosis of MCF-7 cells. Through analysis of RNA sequencing data quality and differentially expressed genes (DEGs), 67 significantly downregulated genes and 260 significantly upregulated genes were identified to be involved in OA treatment of MCF-7 cells. Among these genes, 43 unique DEGs were enriched in several signaling pathways and Gene Ontology terms, such as p53 signaling pathway, TNF signaling pathway and mTOR signaling pathway. Six downregulated genes, including THBS1, EDN1, CACNG4, CCN2, AXIN2 and BMP4, as well as six upregulated genes, including ATF4, SERPINE1, SESN2, PPARGC1A, EGR1 and JAG1, were selected as target genes in response to OA treatment. The inhibitory effect of OA on breast cancer was also found in the following mouse experiments. Our study provides evidence and molecular support for the treatment of breast cancer with OA.

## INTRODUCTION

Oleanolic acid (OA) is an oleanolic pentacyclic triterpene that was first isolated from the leaves of *Olea europaea L.* by F.B. Powers in 1908. OA is widely distributed in nature and is an important component of plants. OA is the active ingredient of many Chinese herbal medicines, such as *Olea europaea L*, *Ligustrum Lucidum Ait*, *Swertia millensis*, *Hemsleya chinensis cogn*, etc. OA plays a very special role in the process of plant growth, such as disease prevention, insect prevention and moisture retention [1, 2]. As a traditional Chinese medicine, OA has been used in the treatment of chronic hepatitis and liver injury [3, 4]. In recent years, an increasing number of studies on the biological activity of OA have been performed. Due to its nontoxicity and significant efficacy, OA has been applied to the treatment of many diseases, especially diabetes and cancer [5, 6].

Previous studies have shown that OA can reduce the blood glucose level of diabetic mice and rats. On the one hand, OA can reduce blood glucose by inhibiting the transport of glucose from the stomach to the small intestine and the transport of glucose in small intestinal villi [7, 8]. On the other hand, OA can promote the secretion of insulin and inhibit the formation of pentoside and hydroxymethyl lysine [9, 10]. OA has obvious anti-inflammatory activity, and its function has been reported in arthritis and encephalomyelitis [11–15]. Based on *in vitro* experiments, OA has been confirmed to have antitumor functions in the occurrence and development of a variety of cancers, such as liver cancer, colorectal cancer, lung cancer, ovarian cancer and breast cancer, mainly through inhibiting cell proliferation, promoting cell apoptosis, and inhibiting tumor cell invasion and angiogenesis [16–19]. The migration of MDA-MB-231 human breast cancer cells can be induced by OA through the activation of EGF receptors and MAP kinases [20]. The high salt-induced exaggeration of Warburg-like metabolism can be inhibited by OA in breast cancer cells [21]. Synthetic OA derivatives, including SZC015 and SZC017, have the ability to induce both apoptosis and autophagy in MCF-7 breast cancer cells [22, 23]. OA was reported to protect against genotoxicity in human breast cancer and inhibit the proliferation of highly invasive breast cancer cells by decreasing oxidative stress and oxidative damage to DNA [24]. Although some existing reports have pointed out the roles of OA in breast cancer, its deep molecular mechanism is not clear. Therefore, it is very important to study the transcriptomics of breast cancer under OA treatment.

## MATERIALS AND METHODS

### Materials

Oleanolic acid was extracted from *Ligustrum lucidum* by ultrasonic-assisted technology. Dulbecco’s modified Eagle’s medium (DMEM) was obtained from biological industries (Shanghai, China). A CCK-8 kit, nonessential amino acids (NEAAs), trypsin−EDTA, PBS and penicillin−streptomycin (PS) were obtained from Biosharp (Hefei, China). Cell cycle and apoptosis analysis kits were obtained from Beyotime (Shanghai, China). An Annexin V-FITC/PI apoptosis detection kit was obtained from Vazyme (Nanjing, China).

### Cell culture and treatment

The MCF-10a, 4T1-luc, MDA-MB-231 and MCF-7 cell lines used in this study were donated by Prof. Yong Li and Shoudong Ye (Anhui University, China). These cells were incubated in DMEM supplemented with 10% FBS, 100 units/mL penicillin-streptomycin (PS) and 0.1 mM NEAA at 37°C in a humidified atmosphere of 95% air and 5% CO_2_. Cells were seeded in 6-well plates or 96-well plates. After reaching confluence, the cells were exposed to the indicated concentrations of OA for certain periods of time. Finally, cells were harvested for the detection of cell proliferation and other analyses.

### Cell proliferation assay

Cell viability was measured using a CCK-8 kit (Biosharp, BS350A, Hefei, China). Briefly, cells were seeded in 96-well plates. After the cells adhered to the wall for 12 hours, cells were exposed to 40 μM and 80 μM OA for various hours (0 h, 4 h, 6 h, 8 h, 16 and 24 h), then the supernatant was discarded, and the cells were rinsed with phosphate-buffered saline (PBS). The cells were incubated with CCK-8 for 1 h. Cell viability was determined at the optical density (OD) at 450 nm using the Thermo Scientific^™^ Varioskan^™^ LUX tool (Invitrogen).

### Cell cycle analysis

Cells were treated with 40 μM OA for various hours (0 h, 4 h and 6 h). The cells were then harvested with a trypsin−ethylene diamine tetraacetic acid (T-EDTA) solution, washed twice with PBS and fixed in 70% ethanol for 16 h at 4°C. Fixation was followed by washing twice with PBS. The cells were then stained with a cell cycle and apoptosis analysis kit (Beyotime, C1052) for 30 min at room temperature in the dark and subjected to flow cytometric analysis of DNA content using a FACSCalibur flow cytometer (BD, USA) (excitation wavelength 488 nm). Approximately 10,000 cells were made for each sample. The relative proportion of cells in G0/G1, S and G2/M phases was calculated by FlowJo software.

### Cell apoptosis analysis

Cells were treated with 40 μM OA for various hours (0 h, 4 h and 6 h). The cells were then harvested with a T-EDTA solution, washed twice with PBS and prepared with 1x binding buffer. The cells were then stained for 10 min at room temperature in the dark with Annexin V−FITC/PI double staining with the Annexin V-FITC/PI Apoptosis Detection Kit (Vazyme, A21101). The cells were then analyzed by the FACSCalibur (BD, USA). Approximately 10,000 counts were made for each sample. The percentage of cells undergoing apoptosis was calculated using FlowJo version 10 software.

### Animal experiments

Ten 4-week-old female BALB/c mice were purchased from Anhui Medical University and isolated for one week in the experimental animal room of Anhui University. Then, they were randomly divided into a normal group (CK group, *n* = 5) and an experimental group (OA group, *n* = 5). The luciferase stable transfer cell line 4T1-luc cells was injected subcutaneously into 5~6 intercostals and the lateral abdominal wall of the chest wall of five-week-old female BALB/c mice. The diet of the mice and the growth of the tumor mass at the inoculation site were observed every day. Intragastric administration was started once a day two days after the occurrence of nodules. The OA group received OA (30 mg/kg) drug treatment, and the CK group received equal doses of drug solvent (corn oil). The weight of the mice was recorded every day, and the tumor size was measured with a Vernier caliper. After 14 days of gavage, the bioluminescence intensity of breast cancer in mice was observed by a Tanon-5200 Multifluorescence imaging analysis system. Then, the mice were killed by spinal dislocation, and the tumors were stripped and weighed.

### RNA sequencing and data analysis

RNA sequencing was performed on four groups of cell samples including two controls (CK1 and CK2) and two 40 μM OA-treated samples for 6 h (OA1 and OA2), based on the Illumina HiSeq X10 platform by Personal Biotechnology (Shanghai, China). The raw data were preprocessed by the regular protocol. DESeq2 was used to identify the differentially expressed genes (DEGs) [25]. The Database for Annotation, Visualization and Integrated Discovery (DAVID) v6.8 was employed to select the enriched KEGG pathways and Gene Ontology (GO) terms [26, 27].

### Statistical analysis

All experiments were performed at least three times, and the results are expressed as the mean ± SD. The results were analyzed by one-way ANOVA followed by a SNK-q test for multiple comparisons. All analyses were performed by using SPSS 20.0 software. Data were considered statistically significant with a *p* value less than 0.05.

## RESULTS AND DISCUSSION

### Effects of OA on the viability of breast tumor cells

**Figure 1:**
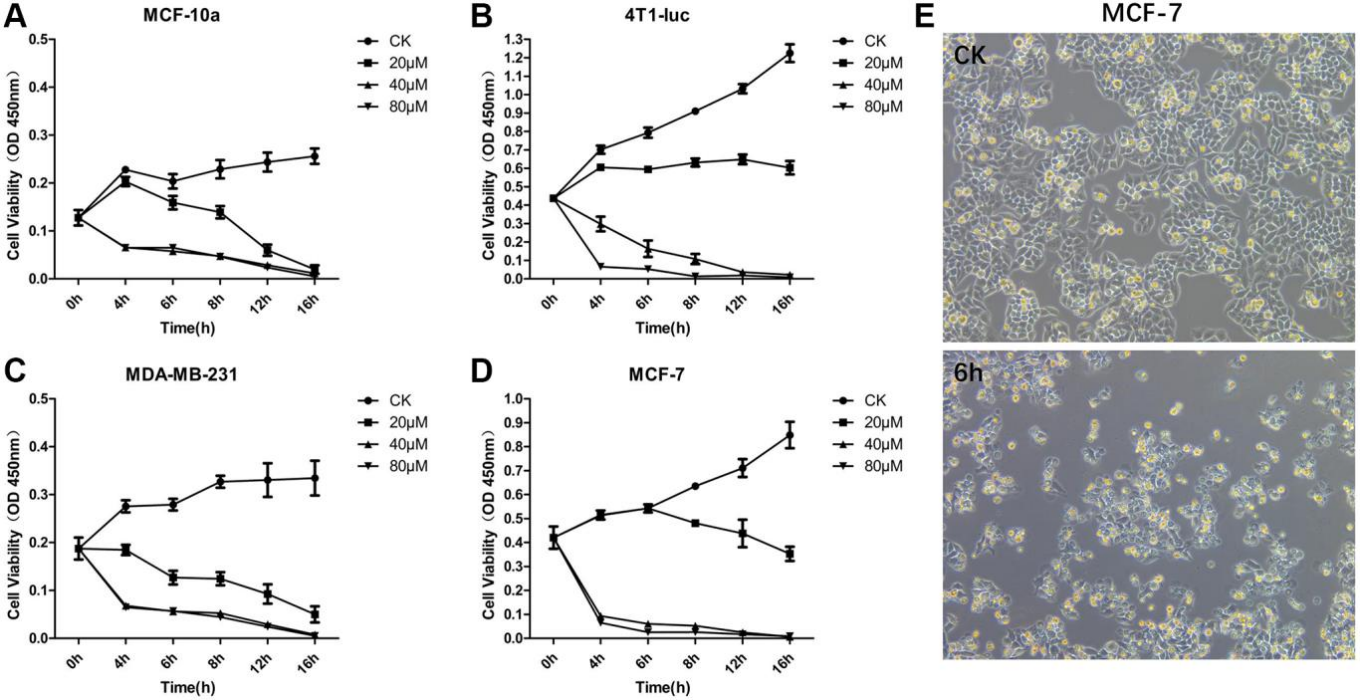
**OA inhibited the cell proliferation of MCF-7 Cells.** MCF-10a (**A**), 4T1-luc (**B**), MDA-MB-231 (**C**) and MCF-7 (**D**) cells were treated with OA in three concentration gradients 20 μM, 40 μM and 80 μM for 16 hours (16 h), and the cell activity of each group was then detected by CCK-8. MCF-7 cells were observed at 6 hours after OA treatment and in the control group (CK) (**E**). Values are mean ± SD (*n* = 5). The cell viability was observed in OD 450 nm.

To study the effect of different concentrations of OA on the proliferation of breast tumor cells, we set three concentration levels: 20 μM, 40 μM and 80 μM. MCF-10a, 4T1-luc, MDA-MB-231 and MCF-7 cells were treated with the above concentrations for 16 hours (16 h), and the cell activity of each group was then detected by the CCK-8 method (Figure 1A–1D). The results showed that a low concentration of OA (20 μM) had no significant effect on the survival rate of MCF-7 and 4T1-luc cells. When the concentration of OA was 80 μM, the cell survival rate decreased significantly at both 4 h and 16 h after OA treatment, which indicated that a high concentration of OA (80 μM) had severe toxicity. After 40 μM OA treatment, the cell survival rate decreased significantly from 4 h to 16 h, which indicated a more effective drug effect. Compared with that of the control group, the cell survival rate began to decrease after 4 h of 40 μM OA treatment. When 40 μM OA treatment was carried out for 6 h, the cell survival rate decreased to a controllable range, which was the best time to collect samples for RNA sequencing (Figure 1E).

### Effects of OA on cell cycle and apoptosis in MCF-7 cells

**Figure 2:**
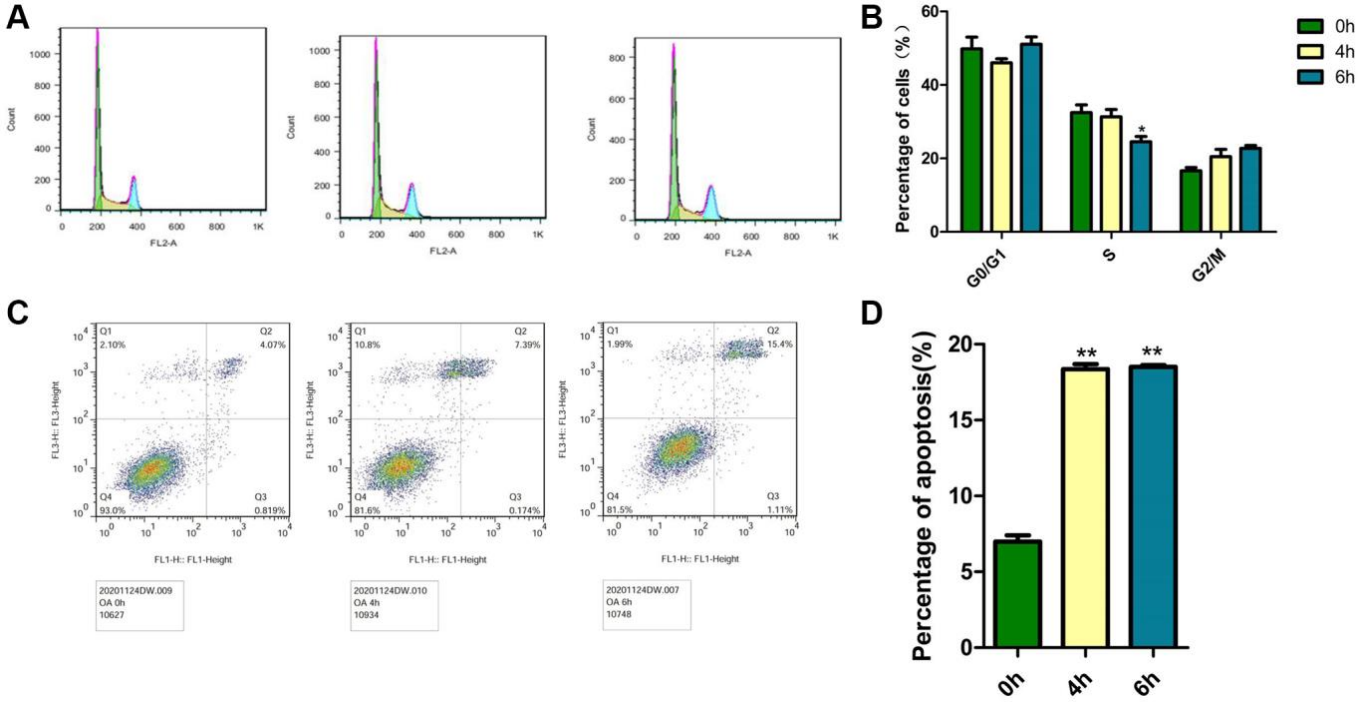
**Effects of OA on the cell cycle and apoptosis in MCF-7 cells.** Cells were treated with 40 μM OA for various hours (0 h, 4 h, 6 h and 8 h). (**A**) PI staining was conducted to observe the effect of OA on the cell cycle *in vitro*. Representative images were shown. (**B**) At least three independent experiments were performed, and the samples were prepared in triplicate. The data represent the mean ± SD. ^*^P < 0.05, compared to control group. (**C**) Annexin V FITC/PI double staining was conducted to observe the effect of OA on apoptosis *in vitro*. Representative images were shown. (**D**) At least three independent experiments were performed, and the samples were prepared in triplicate. The data represent the mean ± SD. ^*^p < 0.05, ^**^P < 0.01, compared to control group.

The decrease in cell viability might be caused by cell cycle arrest or apoptosis. The cell cycle and apoptosis in MCF-7 cells were analyzed by flow cytometry measurements after treatment with 40 μM OA for various hours (0 h, 4 h and 6 h). The cell cycle was divided into the phases of sub-G1(M1), G1/G0 (M2), S (M3) and G2/M (M4) phases by flow cytometry, but in tumor pathobiology, the ratio of S phase cells is usually used as an indicator to judge the proliferative state of tumors. As shown in Figure 2A, when OA was treated for 6 hours, the proportion of S phase was the lowest, which was 24.54%. The data also showed that OA reduced cell proliferation in a time-dependent manner. To further assess the modes of cell apoptosis or necrosis induced by these compounds, MCF-7 cells were treated with 40 μM OA for various hours (0 h, 4 h and 6 h) (Figure 2B). Then, the cells were stained with Annexin V−FITC/PI and analyzed by flow cytometry. There were three subgroups in the experiment. The Annexin V−FITC−/PI− population was considered living cells. The Annexin V−FITC+/PI− population was considered early apoptotic cells, while Annexin V−FITC+/PI+ populations were considered late apoptotic cells. As shown in Figure 2C, cells treated with OA for 4 h were mainly at the early phase of apoptosis, and 6 hours after OA treatment, cells were at the end phase of apoptosis. OA at 40 μM induced significant (^*^p < 0.05) cell apoptosis at 4 h and 6 h (Figure 2D). These data indicate that OA mainly induces MCF-7 cell apoptosis.

The inhibitory effect of OA and its derivatives on breast cancer has been reported in previous studies [28–30]. The semisynthetic analog of OA, the fused hybrids of OA and multiple drugs of ursolic acid (UA) and OA have been shown to inhibit the proliferation and induce apoptosis of breast cancer cells [31–33]. In this study, we also confirmed that OA can inhibit the proliferation and induce the apoptosis of MCF-7 cells. To systematically study the mechanism by which OA inhibits breast cancer, we used high-throughput sequencing technology for transcriptome analysis.

### Data quality of RNA sequencing and differentially expressed gene analysis

**Figure 3:**
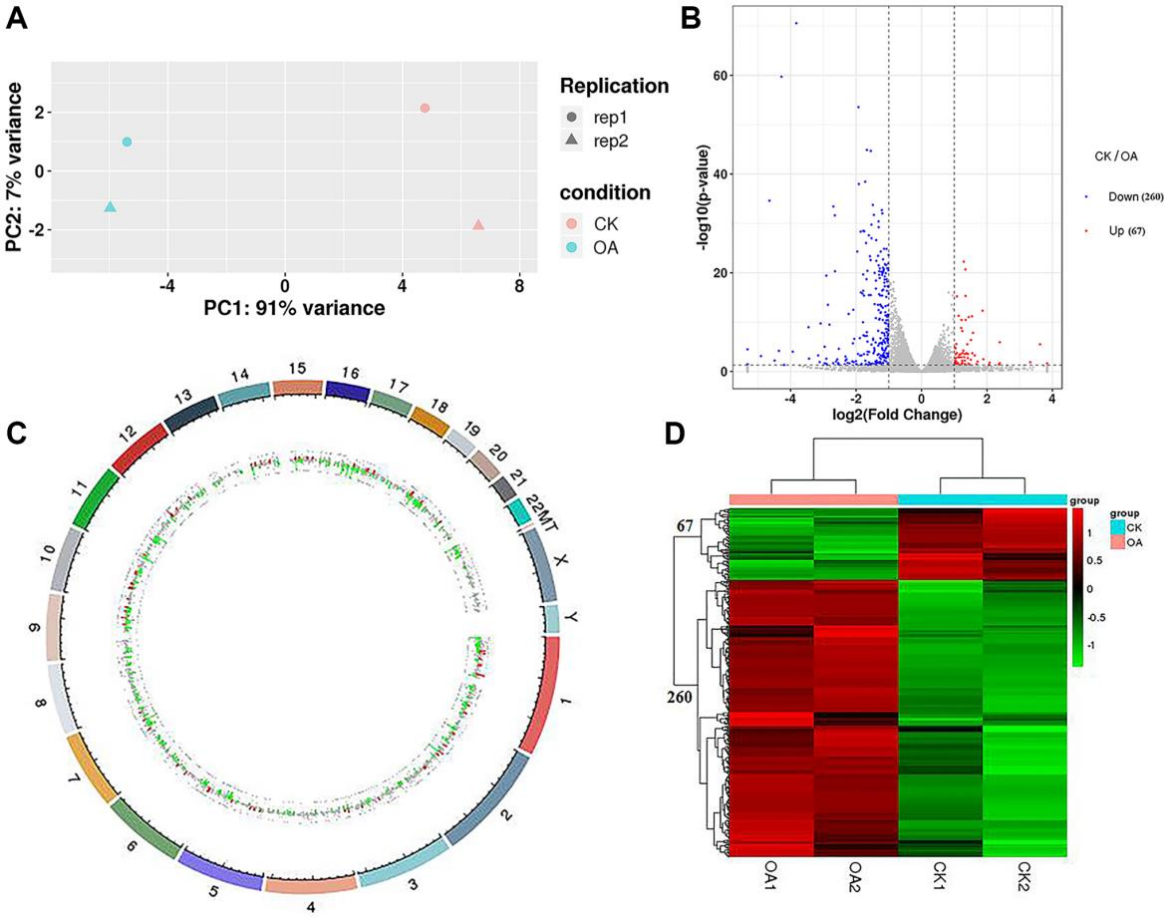
**RNA-seq data analysis.** (**A**) PCA was conducted on samples from four groups (CK1, CK2, OA1 and OA2). (**B**) A volcano plot of 327 DEGs, including 67 downregulated genes and 260 upregulated genes in response to OA treatment was drawn. (**C**) Each DEG was labeled on 23 pairs of chromosomes of the human genome based on the genome location information. (**D**) The Euclidean method was used to calculate the distance and combined with the longest distance method (complete linkage) to conduct two-way hierarchical clustering analysis on the 327 DEGs and the samples of four groups.

Four groups of samples were sequenced to obtain the raw data, and the data quality was evaluated. The results showed that the Q20 values of the four groups of sequencing samples were more than 95%, and the Q30 values were more than 92%, which met the requirements for subsequent data analysis. After data filtering and sequence alignment, we obtained the gene expression profiles of four groups of sequencing samples with a total of 19,970 transcripts. The results of principal component analysis (PCA) showed that the transcriptome data could distinguish the control group (CK1 and CK2) from the treatment group (OA1 and OA2) (Figure 3A).

By comparing the gene expression profile data of the CK group and OA group (CK/OA), based on the threshold standard of |log2 fold change| > 1 and significance level *p*-value < 0.05, we screened 327 differentially expressed genes (DEGs) from 19970 transcriptome annotated genes, including 67 upregulated genes and 260 downregulated genes. The volcano plot of all DEGs is shown in Figure 3B. The details of these DEGs are shown in Supplementary Table 1. According to the genome location information, each DEG was labeled on the human genome (Figure 3C). DEGs were distributed on 23 chromosomes of the human genome, the most of which were the first chromosome, at approximately 42/327. There were also three differentially expressed genes identified on chromosome X: TSC22D3, REPS2 and CLCN5. According to the expression level of the same gene in different samples and the expression pattern of different genes in the same sample, the Euclidean method was used to calculate the distance, and combined with the longest distance method (complete linkage) to conduct two-way hierarchical clustering analysis on the selected differentially expressed genes and each group of samples. As a result, the four groups of samples were significantly distinguished by 67 upregulated gene sets and 260 downregulated gene sets by comparison of CK/OA. The heatmap of clusters is shown in Figure 3D.

For the differentially expressed genes screened above, we ranked them by the |log2 fold change| value to identify the top 10 downregulated genes or upregulated genes in response to OA treatment, which are listed in Table 1. The top 10 downregulated genes were KCND3 (potassium voltage-gated channel subfamily D member 3), PZP (PZP alpha-2-macroglobulin like), EDN2 (endothelin 2), FOXD2 (forkhead box D2), EDAR (ectodysplasin A receptor), ABCA1 (ATP binding cassette subfamily A member 1), MISP3 (MISP family member 3), POU3F2 (POU class 3 homeobox 2), MYLIP (myosin regulatory light chain interacting protein) and CLDND2 (claudin domain containing 2). The top 10 upregulated genes were IL1RN (interleukin 1 receptor antagonist), TRPV3 (transient receptor potential cation channel subfamily V member 3), TNFRSF14 (TNF receptor superfamily member 14), CDH15 (cadherin 15), IFITM10 (interferon induced transmembrane protein 10), CHAC1 (ChaC glutathione specific gamma-glutamylcyclotransferase 1), MARVELD3 (MARVEL domain containing 3), NFATC4 (nuclear factor of activated T cells 4), GDF15 (growth differentiation factor 15) and CAVIN4 (caveolae associated protein 4).

**Table 1:** The top 10 up-regulated or down-regulated genes based on ranking of expression changes.

**Ensembl ID**	**Gene Name**	**Gene Description**	**Mean_OA**	**Mean_CK**	**log2FC**	***p* value**
**Down (OA/CK)**						
ENSG00000171385	KCND3	potassium voltage-gated channel subfamily D member 3	2.84	34.95	−3.62	3.06E-06
ENSG00000126838	PZP	PZP alpha-2-macroglobulin like	4.29	42.91	−3.32	1.30E-02
ENSG00000127129	EDN2	endothelin 2	17.90	93.87	−2.39	1.15E-06
ENSG00000186564	FOXD2	forkhead box D2	2.88	15.07	−2.39	2.12E-02
ENSG00000135960	EDAR	ectodysplasin A receptor	2.43	12.50	−2.36	3.90E-02
ENSG00000165029	ABCA1	ATP binding cassette subfamily A member 1	6.74	28.45	−2.08	1.57E-02
ENSG00000141854	MISP3	MISP family member 3	3.37	14.02	−2.06	4.62E-02
ENSG00000184486	POU3F2	POU class 3 homeobox 2	8.68	32.12	−1.89	3.27E-03
ENSG00000007944	MYLIP	myosin regulatory light chain interacting protein	58.79	214.03	−1.86	4.83E-13
ENSG00000160318	CLDND2	claudin domain containing 2	8.74	28.59	−1.71	1.01E-02
**Up (OA/CK)**						
ENSG00000136689	IL1RN	interleukin 1 receptor antagonist	21.04	0.53	5.32	3.24E-05
ENSG00000167723	TRPV3	transient receptor potential cation channel subfamily V member 3	15.37	0.51	4.91	7.50E-04
ENSG00000157873	TNFRSF14	TNF receptor superfamily member 14	336.85	13.44	4.65	2.58E-35
ENSG00000129910	CDH15	cadherin 15	11.51	0.51	4.49	5.95E-03
ENSG00000244242	IFITM10	interferon induced transmembrane protein 10	31.85	1.55	4.36	6.46E-05
ENSG00000128965	CHAC1	ChaC glutathione specific gamma-glutamylcyclotransferase 1	696.01	35.84	4.28	1.94E-60
ENSG00000140832	MARVELD3	MARVEL domain containing 3	9.70	0.53	4.20	4.55E-02
ENSG00000100968	NFATC4	nuclear factor of activated T cells 4	23.52	1.54	3.94	9.66E-05
ENSG00000130513	GDF15	growth differentiation factor 15	996.48	70.30	3.83	2.80E-71
ENSG00000170681	CAVIN4	caveolae associated protein 4	62.95	5.74	3.45	1.07E-09

### Functional enrichment analysis

**Figure 4:**
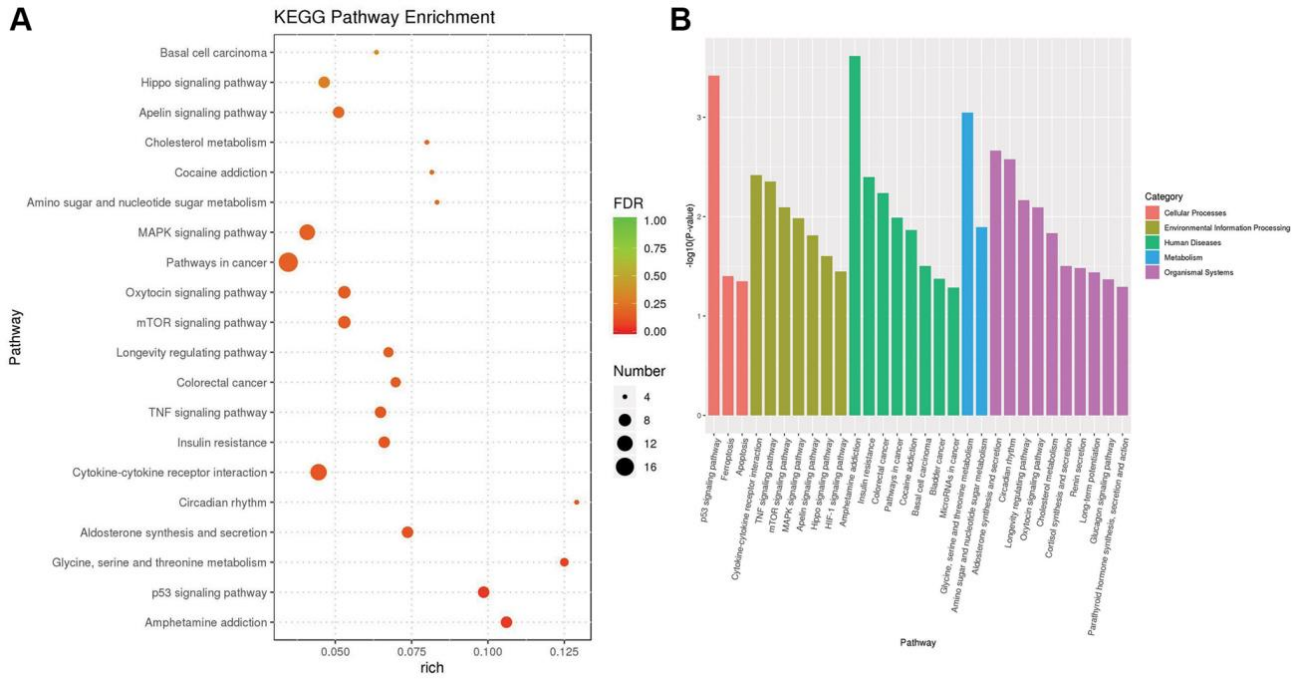
**The results of KEGG pathway enrichment.** (**A**) The plot shows the top 20 significantly enriched KEGG pathways in response to OA treatment in MCF-7 cells. (**B**) The top 20 significantly enriched KEGG pathways were clustered into five pathway categories: cellular processes (red), environmental information processing (green yellow), human diseases (green), metabolism (blue) and organismal systems (purple).

Based on the KEGG pathway database and the method of gene set enrichment analysis (GSEA), we identified 28 KEGG pathways with significant enrichment (*p* value less than 0.05) of differentially expressed genes and selected the top 20 pathways with the lowest *p* value, i.e., the most significant enrichment. The results are shown in Figure 4. Among these results, the cellular processes-related pathways include the p53 signaling pathway, ferroptosis and apoptosis. The related pathways of environmental information processing include the cytokine-cytokine receptor interaction pathway, TNF signaling pathway, mTOR signaling pathway, MAPK signaling pathway, Apelin signaling pathway, Hippo signaling pathway and HIF-1 signaling pathway. Human diseases-related pathways include amphetamine addiction, insulin resistance, colorectal cancer, pathways in cancer, cocaine addiction, basal cell carcinoma, bladder cancer and microRNAs in cancer. Metabolic pathways include glycine, serine and threonine metabolism, amino sugar and nucleotide sugar metabolism. The associated pathways of organic systems include aldosterone synthesis and secretion, circadian rhythm, longevity regulating pathway, oxytocin signaling pathway and cholesterol metabolism, cortisol synthesis and secretion, renin secretion, long-term potentiation, glucagon signaling pathway and parathyroid hormone synthesis, secretion and action.

**Figure 5:**
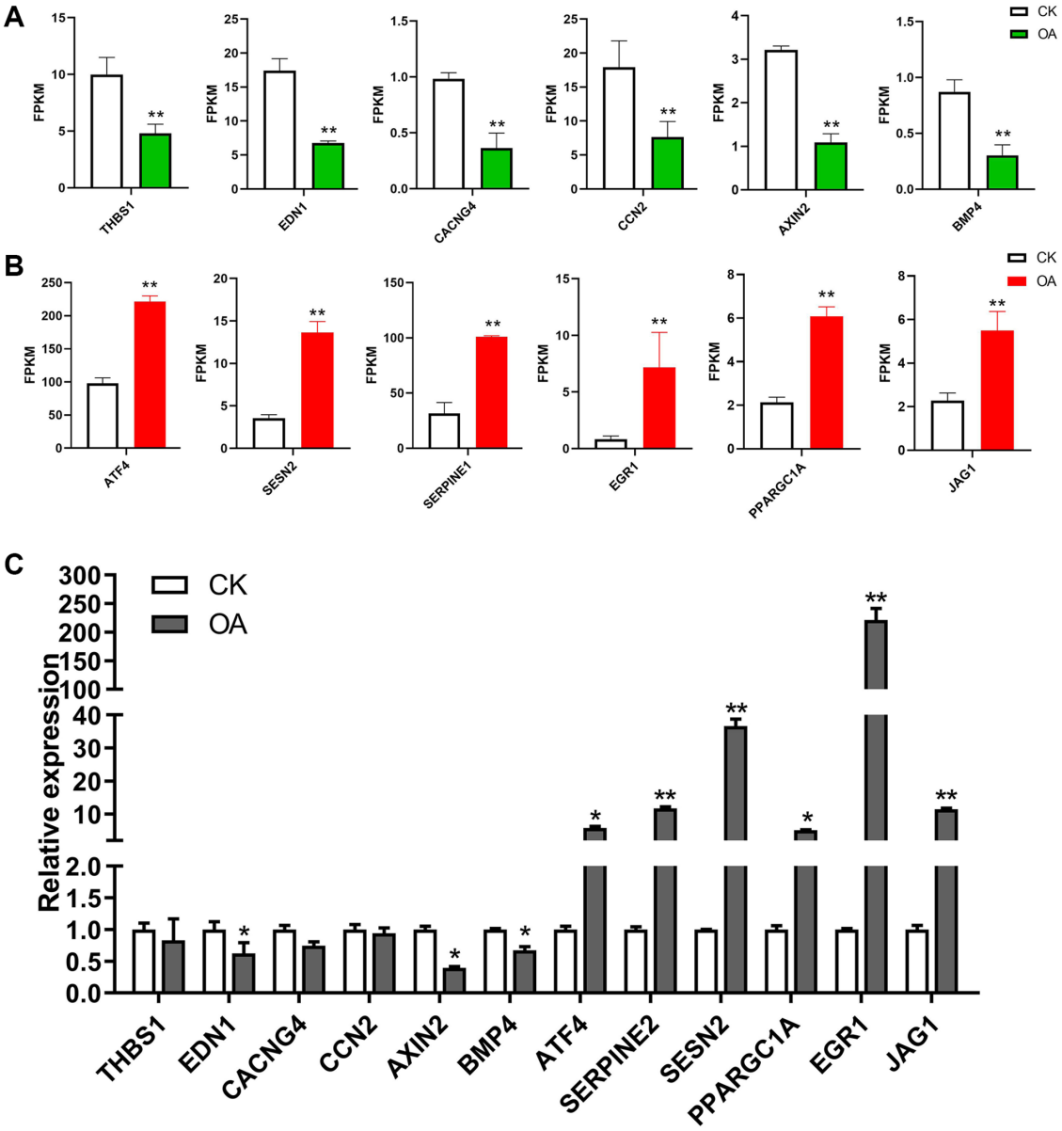
**The regulation of target genes in response to OA treatment.** (**A**) The bar plots show the expression values of six downregulated genes in RNA-seq data, including THBS1, EDN1, CACNG4, CCN2, AXIN2 and BMP4. (**B**) The bar plots show the expression values of six upregulated genes in RNA-seq data, including ATF4, SERPINE1, SESN2, PPARGC1A, EGR1 and JAG1. (**C**) The bar plots show the expression values of above 12 genes based on qPCR results.^*^p < 0.05, ^**^p < 0.01.

Furthermore, the significantly enriched signaling pathways and involved genes are listed in Table 2. In total, there were 43 unique DEGs, including 37 upregulated genes and 6 downregulated genes in these signaling pathways in response to OA treatment. The expression patterns of selected target genes are shown in Figure 5. Six downregulated genes, including thrombospondin 1 (THBS1), endothelin 1 (EDN1), calcium voltage-gated channel auxiliary subunit gamma 4 (CACNG4), cellular communication network factor 2 (CCN2), axin 2 (AXIN2) and bone morphogenetic protein 4 (BMP4), as well as six upregulated genes, including activating transcription factor 4 (ATF4), serpin family E member 1 (SERPINE1), sestrin 2 (SESN2), PPARG coactivator 1 alpha (PPARGC1A), early growth response 1 (EGR1) and jagged canonical Notch ligand 1 (JAG1), were identified in signaling pathways in response to OA treatment. THBS1 has been reported to be involved in breast cancer progression regulated by estrogen [34]. The critical roles of EDN1 in the proliferation and migration of human breast cancer cells have been demonstrated in a previous study [35]. CACNG4 has been reported to regulate tumor cell growth and dissemination by altering calcium signaling in breast cancer [36]. An increase in CCN2 expression levels has been shown to play an important role in the invasion and metastasis of breast cancer [37]. The polymorphisms rs11079571 and rs3923087 of the AXIN2 gene were reported to be associated with an increased risk of breast cancer [38]. The metastasis of breast cancer can be suppressed by activating the canonical BMP4-SMAD7 signaling axis [39]. In estrogen receptor (ER)-negative breast cancer, it has been indicated that ATF4 can activate the expression of the oncogene PSAT1 and promote cell proliferation [40]. Knockdown of SERPINE1 was found to significantly inhibit cell survival and induce apoptosis of breast cancer cells *in vitro* [41]. SESN2 is involved in the suppression of genesis of breast cancer genesis through its interaction with AMP-activated protein kinase (AMPK) [42]. The migration and invasion of breast cancer cells could be promoted by PPARGC1A [43]. Previous studies have demonstrated that EGR1 could suppress the proliferation and migration of breast cancer cells [44, 45]. The overexpression of JAG1 has been observed in breast cancer patients and is associated with the development of distant metastasis [46].

**Table 2:** Significantly enriched signaling pathways and involved genes.

**Pathway**	**Level**	***P* value**	**Up_genes**	**Down_genes**
p53 signaling pathway	Cell growth and death	3.82E-04	BBC3, GADD45A, CDKN1A, PMAIP1, SERPINE1, SESN2	THBS1
TNF signaling pathway	Signal transduction	4.43E-03	LIF, ATF4, CXCL2, PTGS2, CREB5, JAG1	EDN1
mTOR signaling pathway	Signal transduction	8.08E-03	RRAGC, SESN2, FNIP2, SLC3A2, ULK1, FNIP1, FLCN, GRB10	/
Oxytocin signaling pathway	Endocrine system	8.08E-03	PRKAG2, CDKN1A, KCNJ2, NFATC4, CACNA1C, CALML6, PTGS2	CACNG4
MAPK signaling pathway	Signal transduction	1.04E-02	DDIT3, DUSP1, PTPRR, CACNA1C, DUSP4, DUSP10, ATF4, AREG, GADD45A, DUSP5, CSF1R	CACNG4
Apelin signaling pathway	Signal transduction	1.55E-02	PRKAG2, PPARGC1A, EGR1, CALML6, SERPINE1, JAG1	CCN2
Hippo signaling pathway	Signal transduction	2.49E-02	SNAI2, BBC3, AREG, SERPINE1	AXIN2, CCN2, BMP4
HIF-1 signaling pathway	Signal transduction	3.56E-02	CDKN1A, HMOX1, HKDC1, SERPINE1	EDN1
Glucagon signaling pathway	Endocrine system	4.29E-02	PRKAG2, PPARGC1A, ATF4, CALML6, CREB5	/

**Figure 6:**
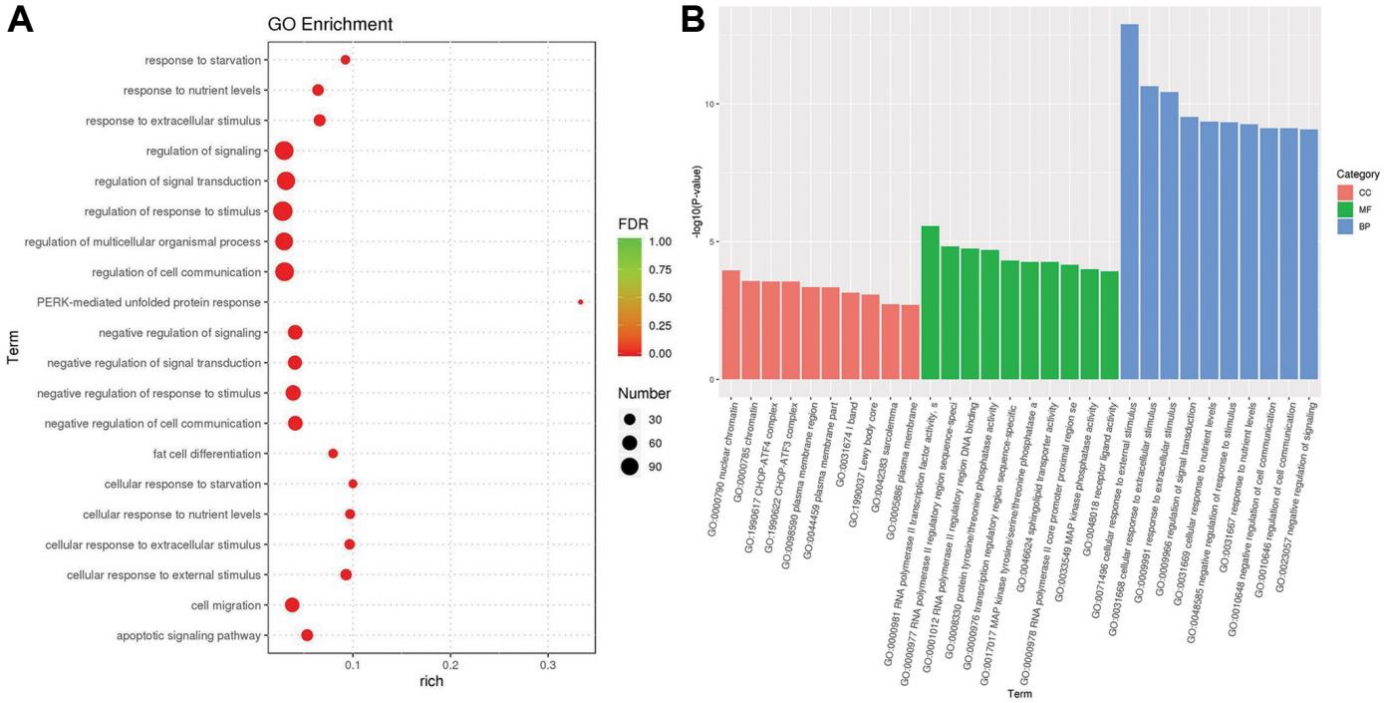
**The results of gene ontology enrichment**. (**A**) The plot shows the top 20 significantly enriched gene ontology (GO) terms in response to OA treatment in MCF-7 cells. (**B**) The top 10 significantly enriched GO terms in three distinct functional groups, including cellular component (CC) colored red, molecular function (MF) colored green and biological process (BP) colored blue.

We used the topGO tool to perform Gene Ontology (GO) enrichment analysis. In the analysis, we used the GO terms of the different genes to obtain the gene list and the number of genes enriched in each term, and then calculated the *p* value (*p* value < 0.05) by the hypergeometric distribution method to identify the GO terms in which the different genes were signaling enriched compared with the whole genome background in order to determine the main biological functions of different genes. Finally, we obtained 1489 GO terms of biological process (BP), 110 GO terms of cellular component (CC) and 225 GO terms of molecular function (MF). According to the GO enrichment results, we selected the top 20 GO terms with the lowest false discovery rate (FDR) value, that is, the most significant enrichment, for display with the degree of enrichment measured by rich factor and the number of genes enriched in each GO term. The bubble chart of the top 20 enriched GO terms is shown in Figure 6A. Furthermore, we selected the top 10 GO terms with the lowest *p* value, i.e., the most significant enrichment, from each GO category for display. The bar chart of the top 10 enriched GO terms is shown in Figure 6B. The most significant functional enrichment categories were GO:0000790 nuclear chromatin of CC, GO:0000981 RNA polymerase II transcription factor activity, sequence-specific DNA binding of MF, and GO:0071496 cellular response to external stimulus of BP.

### OA inhibited the breast tumor growth in mice

**Figure 7:**
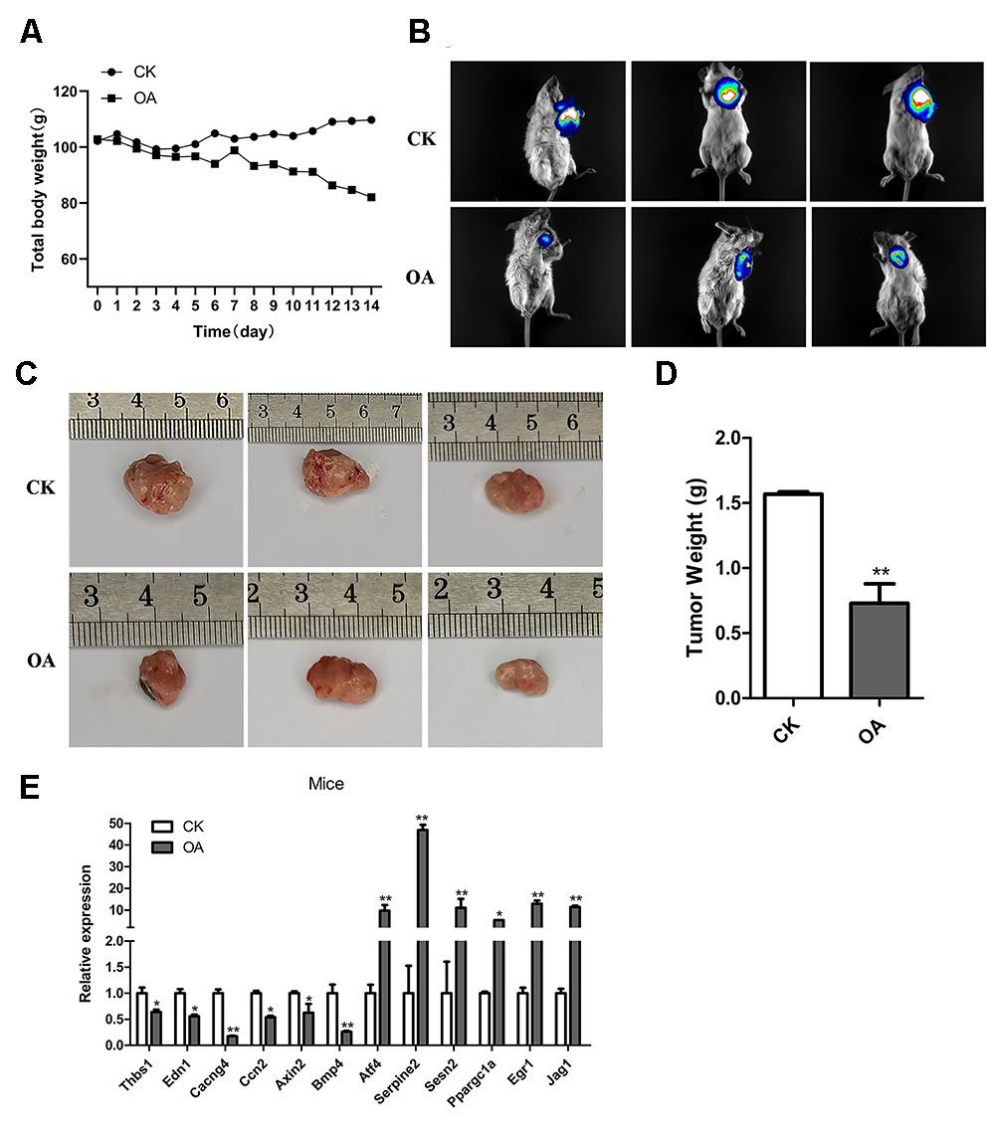
**OA inhibited the breast tumor growth in mice.** (**A**) The total body weights of five mice were compared between the CK group (*n* = 5) and OA group (*n* = 5) every day during the feeding process. (**B**) The bioluminescence intensity of breast tumors in three CK mice and three OA mice was observed by a Tanon-5200 Multifluorescence imaging analysis system. (**C**) The tumor sizes of three CK mice and three OA mice were measured by rulers. (**D**) The tumors from the CK group and OA group were weighed and compared (*n* = 5), ^**^P < 0.01. (**E**) Validation of target gene expression in mice from the CK group and OA group tested by qPCR, ^*^p < 0.05, ^**^P < 0.01.

To further explore the anti-cancer effect of OA, we carried out *in vivo* experiments in mice. After transplantation of tumor cells, mice were administered OA for two weeks. *In vivo* bioluminescence imaging was used to explore the effect of OA on tumors in mice. As a result, compared with CK tumor model mice, the body weight of OA group mice decreased to some extent, while the size and weight of tumor decreased significantly. (Figure 7A–7D). It showed that OA might inhibit the breast tumor growth in mice, although OA treatment would also affect other physiological indexes of mice. According to the six upregulated genes and six downregulated genes identified by the above cell-level RNA-seq analysis, the function was verified by qPCR *in vivo,* the results of which were consistent (Figure 7E). The primers of target genes for qPCR were listed in Supplementary Table 2.

In conclusion, both *in vitro* and *in vivo* experiments confirmed that OA had an inhibitory effect on breast tumors. The key genes, including six downregulated genes THBS1, EDN1, CACNG4, CCN2, AXIN2 and BMP4, as well as six upregulated genes ATF4, SERPINE1, SESN2, PPARGC1A, EGR1 and JAG1 associated with OA inhibiting the proliferation of MCF-7 cells, were screened by RNA-seq, and their expression patterns were verified in mouse experiments. The p53 signaling pathway, TNF signaling pathway and mTOR signaling pathway were identified to be involved in the response to OA treatment in breast tumor growth.

## Supplementary Materials

Supplementary Table 1

Supplementary Table 2
