# Supplementary material for: Transcriptome study of oleanolic acid in the inhibition of breast tumor growth based on high-throughput sequencing
Source: Aging (Albany NY). 2021 Oct 4;13(19):22883–97. doi: 10.18632/aging.203582 (PMC8544337; doi:10.18632/aging.203582)
Supplement: Supplementary Table 2 [file aging-13-203582-s002.pdf]

SUPPLEMENTARY TABLES

Please browse Full Text version to see the data of Supplementary Table 1.

Supplementary Table 1. Details of identified DEGs in response to OA treatment in MCF-7 cells.

Supplementary Table 2. Sequences of primers for the genes.

Gene	Sense Primer (5′ to 3′)	Anti-sense Primer (5′ to 3′)
THBS1	TTGTCTTTGGAACCACACCA	CTGGACAGCTCATCACAGGA
Thbs1	CCAAAGCCTGCAAGAAAGAC	CCTGCTTGTTGCAAACTTGA
EDN1	CCAAGGAGCTCCAGAAACAG	GATGTCCAGGTGGCAGAAGT
Edn1	CTGCCAAGCAGGAAAAGAAC	TTGTGCGTCAACTTCTGGTC
CACNG4	ACTGCTTCCGGATCAATCAC	TTCTTGCGGCTGTAGATCCT
Cacng4	AGCCCTGTCGTTTATTGTGG	GACCTTGAACTGGACCTGGA
CCN2	CCGTACTCCCAAAATCTCCA	GTAATGGCAGGCACAGGTCT
Ccn2	CAAAGCAGCTGCAAATACCA	GGCCAAATGTGTCTTCCAGT
AXIN2	CTCCCCACCTTGAATGAAGA	GTTTCCGTGGACCTCACACT
Axin2	TAGGCGGAATGAAGATGGAC	CTGGTCACCCAACAAGGAGT
BMP4	TGAGCCTTTCCAGCAAGTTT	GCCTCCTAGCAGGATGACAG
Bmp4	CGTTACCTCAAGGGAGTGGA	ATGCTTGGGACTACGTTTGG
ATF4	TCAAACCTCATGGGTTCTCC	GTGTCATCCAACGTGGTCAG
Atf4	TCCTGAACAGCGAAGTGTTG	ACCCATGAGGTTTCAAGTGC
SERPINE1	CTCTCTCTGCCCTCACCAAC	GTGGAGAGGCTCTTGGTCTG
Serpine1	ACGTTGTGGAACTGCCCTAC	GCCAGGGTTGCACTAAACAT
SESN2	TGCTGTGCTTTGTGGAAGAC	GCTGCCTGGAACTTCTCATC
Sesn2	TAGCCTGCAGCCTCACCTAT	GATTTTGAGGTTCCGTTCCA
PPARGC1A	CCTTGCAGCACAAGAAAACA	CTGCTTCGTCGTCAAAAACA
Ppargc1a	CCGAGAATTCATGGAGCAAT	TTTCTGTGGGTTTGGTGTGA
EGR1	TGACCGCAGAGTCTTTTCCT	TGGGTTGGTCATGCTCACTA
Egr1	CCACAACAACAGGGAGACCT	ACTGAGTGGCGAAGGCTTTA
JAG1	GACTCATCAGCCGTGTCTCA	TGGGGAACACTCACACTCAA
Jag1	CAGTGCCTCTGTGAGACCAA	AGGGGTCAGAGAGACAAGCA
